# The Potential Role of Iceland in Northern Europe’s Protein Self-Sufficiency: Feasibility Study of Large-Scale Production of Spirulina in a Novel Energy-Food System

**DOI:** 10.3390/foods12010038

**Published:** 2022-12-22

**Authors:** Asaf Tzachor, Catherine E. Richards, Asger Smidt-Jensen, Arnar Þór Skúlason, Alfons Ramel, Margrét Geirsdóttir

**Affiliations:** 1 Centre for the Study of Existential Risk, University of Cambridge, Cambridge CB2 1SB, UK; 2 School of Sustainability, Reichman University, Herzliya 4610101, Israel; 3 Department of Engineering, University of Cambridge, Cambridge CB2 1PZ, UK; 4 Centre for Food Technology, Danish Technological Institute (DTI), 8000 Århus, Midtjylland, Denmark; 5 Faculty of Life and Environmental Sciences, University of Iceland, Ssn. 600169-2039, 113 Reykjavík, Iceland; 6 Faculty of Food Science and Nutrition, University of Iceland, Ssn. 600169-2039, 113 Reykjavík, Iceland; 7 Bioactive Compounds Group, Matís, 113 Reykjavík, Iceland

**Keywords:** protein, dependency, self-sufficiency, algae, Spirulina, food security, Iceland, Europe

## Abstract

Europe is dependent on protein-rich crop imports to meet domestic food demand. This has moved the topic of sustainable protein self-sufficiency up the policy agenda. The current study assesses the feasibility of protein self-sufficiency in Iceland, and its capacity to meet Northern Europe’s demand, based on industrial-scale cultivation of Spirulina in novel production units. Production units currently operating in Iceland, and laboratory-derived nutritional profile for the Spirulina cultivated, provide the basis for a theoretical protein self-sufficiency model. Integrating installed and potentially installed energy generation data, the model elaborates six production scale-up scenarios. Annual biomass produced is compared with recommended dietary allowance figures for protein and essential amino acids to determine whether Northern Europe’s population demands can be met in 2030. Results show that Iceland could be protein self-sufficient under the most conservative scenario, with 20,925 tonnes of Spirulina produced using 15% of currently installed capacity. In a greater allocation of energy capacity used by heavy industry, Iceland could additionally meet the needs of Lithuania, or Latvia, Estonia, Jersey, Isle of Man, Guernsey, and Faroe Islands. Under the most ambitious scenario utilizing planned energy projects, Iceland could support itself plus Denmark, or Finland, or Norway, or Ireland with up to 242,366 tonnes of biomass. On a protein-per-protein basis, each kilogram of Spirulina consumed instead of beef could save 0.315 tonnes CO_2_-eq. Under the most ambitious scenario, this yields annual savings of 75.1 million tonnes CO_2_-eq or 7.3% of quarterly European greenhouse gas emissions. Finally, practicalities of production scale-up are discussed.

## 1. Introduction

Proteins are an indispensable component of a healthy human diet, and global food security. Specifically, complete proteins that contain the nine essential amino acids (EAAs). Proteins play a fundamental role in meeting metabolic demands and maintaining normal body composition and function [1]. Contemporarily, animal source foods (ASF), including eggs, milk, beef, pork, poultry and fish, are the predominant sources of complete protein in human nutrition [2,3,4].

However, a reliance on conventional terrestrial and aquatic ASF as protein sources has substantial environmental implications. For instance, globally, these food systems account for over 25% of greenhouse gas (GHG) emissions and 70% of freshwater withdrawals, place a burden on 50% of global land and cause 78% of marine and freshwater eutrophication [5,6,7]. It has been calculated that producing a kilogram of meat from beef cattle emits 99.48 kg CO_2_-eq, with alternatives, such as mutton meat, pig meat and poultry meat, emitting 39.72 kg CO_2_-eq, 12.31 kg CO_2_-eq and 9.87 kg CO_2_-eq, respectively [8]. Aquaculture, which contributes protein-rich and relatively resource efficient alternatives to terrestrial ASF [9], is associated with other types of environmental pressures, including overfishing, bycatch and habitat degradation [10,11,12].

Considering this, several seminal studies have advocated moving away from conventional ASF farming methods to ensure more sustainable diets [13,14,15]. Sustainable agriculture propositions, particularly for short term food system transitions, include organic or ecological farming where organic fertilizers, such as compost manure, replace synthetic counterparts and environmentally optimized techniques, such as crop rotation and companion planting, are often emphasized [16,17]. Regenerative agriculture, which leverages permaculture principles, is another approach to enhances food production through conservation and rehabilitation activities, such as topsoil regeneration and improved biodiversity [18,19].

In addition to environmental ramifications of ASF, a few regions, including the European Union (EU), depend heavily on importation of feed crops to meet the protein requirements of their local livestock sectors [20,21,22,23,24]. According to the EU, domestic production of protein-rich feed crops is insufficient to meet demand, obligating member countries to import 75% of their protein requirements, including 95% of soy cake consumption [25,26,27]. Dependency on third parties exposes such countries to protein supply chain disruptions, including institutional interventions and disruptions such as those experienced during the COVID-19 pandemic [28,29,30], as well as biotic and abiotic stressors, including pests, pathogens and alternations in atmospheric and edaphic factors experienced by producers and exporters of high-protein crops [31,32,33]. This renders European food security vulnerable, particularly to the cascading acute and chronic impacts of anthropogenic climate change on global agriculture systems [34].

These concerns have moved the topic of sustainable protein self-reliance higher up the policy agenda in Europe. Initiatives such as the EU Protein Plan aim to incentivize and accelerate the development of plant proteins [35], while complementary studies suggest alternative food sources that may be cultivated sustainably, within the European region [20,24,27,36,37]. In particular, novel ‘future food’ protein sources produced in adaptable, often closed environment, configurations have been proposed as substitutions for conventional meat. Cellular agriculture, including cultured meat and dairy, microbial food culture, including mycoprotein, insect and algae based foods, currently under-development, have the potential to reduce environmental impacts of conventional ASF by some 80%. [38,39,40,41]. Spirulina blue green algae (*Arthrospira platensis*), is one such ‘future food’ garnering strong interest as a nutritious, sustainable and risk-resilient alternative [42,43,44].

Accordingly, this study aims to add to an expanding and consequential literature on protein self-sufficiency in Europe, with a focus on Iceland, considering Spirulina as an alternative protein source (see Section 2.1: system boundaries). It has two objectives, addressed using an empirical data-based modelling methodology. First, this study assesses the feasibility of protein self-sufficiency in Iceland based on industrial-scale production of Spirulina in a novel, industrial-scale, biomass cultivation system powered by renewable energy currently in operation (hereafter referred to as the Hellisheidi production unit, PU). Second, it evaluates Iceland’s potential role in supporting other countries in Northern Europe to achieve protein self-sufficiency under six Spirulina production scale-up scenarios.

## 2. Methods and Materials

This section summarizes the system boundaries (Section 2.1), protein self-sufficiency model design (Section 2.2), input data related to Spirulina cultivation in the Hellisheidi PU (Section 2.3) and renewable energy sources (Section 2.4) in Iceland, Spirulina production-scale up scenarios (Section 2.5) and protein and EEA dietary requirements and population demand for Northern European countries (Section 2.6), underpinning this feasibility assessment. Full documentation, including input data and calculations, is provided in the Supplementary Information (Supplementary Information S1).

### 2.1. System Boundaries

Iceland was selected as the focus of this protein self-sufficiency feasibility assessment for two main reasons. First, the country is integrated with the EU through participation in the single market. Specifically, it is a partner in the EU’s Northern Dimension framework promoting cooperation in Northern Europe, the region including the British Isles, the Nordic countries and the Baltic states. Iceland is representative of European-wide protein self-sufficiency concerns given its current reliance on high-protein feed crop imports, with exploitative ocean fisheries as the alternative [45,46]. Both options entail food security and ecological complications. While domestic livestock provides approximately 90% of meat, 96% of eggs and 99% of dairy products, this agricultural sector depends on the import of feed sources. Similarly, the fishing industry if highlight dependent on fuel imports [45].

Second, over 99% of total electricity in Iceland is generated from low-carbon, renewable and non-intermittent hydropower and geothermal resources [47]. The Icelandic energy system could potentially support novel, reliable and food production unit configurations. For instance, where alternative proteins are derived from phototrophic organisms (algae) cultivated in closed artificial photosynthesis systems [48,49], including in controlled bioreactors [38,50] and photobioreactors (PBRs) [51,52,53,54].

This study assesses the industrial production of Spirulina in Iceland as a method to achieve protein self-sufficiency for technical practicality and data availability reasons. First, this edible, microscopic, filamentous cyanobacterium is recognized as a superb provider of complete protein [55], with nutritional benefits including antioxidant and anti-inflammatory effects [56]. It is considered a good alternative food source to conventional ASF by dint of its fast growth rate, digestibility (namely, lack of a cellulose cell wall) and chemical composition, which, in addition to EAAs, includes carbohydrates, minerals (particularly iron), fatty acids and vitamins [57,58]. Previous studies have reported that Spirulina contains a higher content of protein (up to 70% per 100 g) than meat from beef cattle (up to 30% per 100 g lean meat) [43]. Other analyses have emphasized the reduced environmental impacts associated with Spirulina [59,60,61]. For instance, by replacing beef meat, Spirulina renders feed crop imports for ruminants redundant. Indeed, previous studies have already called to diversify protein production with this autotroph [62,63,64].

Second, Spirulina is already produced successfully in Iceland, meaning that empirical, rather than theoretical, data is available. Furthermore, the authors had access to relevant data repositories and proprietary data from in situ analysis of the Hellisheidi PU currently producing Spirulina in Iceland. This information forms the basis of a protein self-sufficiency model underpinning this study (see Supplementary Information S1).

This feasibility assessment was conducted in the year 2030, which corresponds with the Sustainable Development Goals Agenda and minimizes uncertainty as a year within the next decade.

### 2.2. Model Design

To achieve this study’s objectives, a spreadsheet-based model was built to evaluate the potential of six scenarios of Spirulina production scale-up, using currently installed and potentially installed renewable energy capacity, to meet the projected protein and EAA demand of Icelandic and Northern European populations in 2030.

The protein self-sufficiency model consists of two main components: protein supply, including real-world Spirulina production data, referred to as Component A of the model; and protein demand, accounting for daily protein intake requirements as well as the suitability of Spirulina biomass to satisfy these requirements, referred to as Component B.

On protein supply (model Component A), Section 2.3 describes the cultivation data for the Hellisheidi PU. This facility has attracted scientific interest and received funding from the EU Horizon 2020 Research and Innovation Program, the Icelandic Technology development Fund and the Eurostars-2 Joint Program [65]. Section 2.4 details the data on national installed-capacity and potential-capacity of energy generation projects, obtained from the government of Iceland. These data are combined in Section 2.5 to characterize potential Spirulina production scale-up under six different electricity allocation scenarios, ultimately defining how much high-protein biomass can be supplied in the protein self-sufficiency model.

On protein demand (model Component B), Section 2.6 details the nutritional profile, determined by laboratory analysis, of the Spirulina produced by the renewable-energy-food-biomass-cultivation system in Iceland. It documents the recommended daily allowance (RDA) of protein and EAAs, as specified by the Food and Agriculture Organization (FAO), the World Health Organization (WHO) and the United Nations University (UNU), as well as population projections for Northern European countries. These data are combined to define protein demand, which is reconciled with protein supply, in the protein self-sufficiency model to determine to what extent Spirulina production in Iceland can meet the protein and EEA requirements of Northern European populations (Section 3.1).

In addition to Components A and B, the protein self-sufficiency model underscores ancillary environmental benefits of Spirulina production, namely GHG emissions reduction (see Supplementary Information S1, sheet 3). Based on in situ measurements, previous studies and model assumptions, the protein self-sufficiency model provides a potential range of GHG emissions (in CO_2_-eq) that may be mitigated under each scenario (Section 3.2). For this assessment, global GHG emissions reduction is prioritized over protein self-sufficiency, i.e., the study assumes that Spirulina is incrementally adopted in Western diets as a beef meat protein alternative. For all calculations performed in this study, available open-source data are used.

### 2.3. Spirulina Biomass Production Unit (PU)

Production data from the Hellisheidi PU currently producing Spirulina in Iceland is used as a benchmark for the PUs underpinning the protein self-sufficiency model. This Hellisheidi PU is situated in Hellisheiði geothermal power plant and park, in the Hengill area of southwest Iceland (64°2′12″ N, 21°23′53″ W). It has an installed production capacity of 60 tonnes dry weight (DW) Spirulina (*Arthrospira platensis*) biomass per year.

Spirulina is cultured in a modified Zarrouk medium [66]. The Hellisheidi PU consists of 80 flat panel airlift PBRs, each 180 L in volume. The culture is agitated with pneumatically induced CO_2_-enriched air at flow aeration of 0.5 air volume/medium volume/minute (vvm). Temperatures are kept at 31 ± 2 °C. pH is maintained at 10.8 ± 0.2. Culture is grown under red/blue/UV illumination (USP # 63/026,764) with maximum irradiance of 750 μmol per m^2^s. Light emitting diodes (LEDs) are used for illumination to achieve augmented photosynthesis [67]. Previous analyses have calculated energy-to-light efficiency of LED systems and light-to-algal-biomass conversion efficiency. It was indicated that ±141 kWh is required to cultivate 1 kg of dry weight of algal biomass. This figure corresponds the data retrieved from the Hellisheidi Spirulina PU (139.7 kWh per 1 kg DW). Since only 50% of electrical energy supplied converts into light [68], residual heat must be removed. In the Hellisheidi PU, this is achieved with liquid-liquid heat exchange utilizing cooling pass-through water provided by the geothermal park operations, alongside a stream of geothermal CO_2_ for biofixation in the culture.

Recent research [69] summarized the necessary inputs for Spirulina cultivation and downstream processing in the Hellisheidi PU, including harvest, washing, heating, pasteurization, cooling, parceling and packaging, before the biomass leaves factory gates (see Supplementary Information S1, sheet 1). Electricity for its operation is supplied by connection to ON Power (Orka náttúrunnar, Reykjavik, Iceland) geothermal power station, with an associated GHG emissions intensity of 0.0083 kg CO_2_-eq per kWh [70]. Analyses have also determined water footprint, land footprint, Nitrogen fertilizer, phosphorus fertilizer and iron sulfate, as well as net GHG intensity of the Hellisheidi PU on a functional unit basis. Production of 1 kg DW Spirulina biomass requires 0.0964 m^2^ of marginal lands, 21.4 m^3^ of freshwater, 0.44 kg NaNO_3_, 1.26 kg K_2_HPO_4_, 0.16 kg FeSO_4_, 139.7 kWh of electricity and 1.80 kg of CO_2_.

Accounting for GHG emissions of inputs, including materials and construction, the Hellisheidi PU production process was estimated to be carbon neutral (kg CO_2_-eq per kg DW Spirulina biomass) [69]. In this study, the modelled PUs are assumed connected to the national electric grid, as opposed to ON Power geothermal power station. Therefore, a different GHG footprint per unit of electricity production was adopted: 0.0036 g CO_2_-eq per kWh, based on Landsvirkjun National Power Company, Iceland, data [71]. Using this figure, the net GHG intensity of the PUs modelled in this study is −0.7086 kg CO_2_-eq per kg DW Spirulina biomass.

### 2.4. Iceland’s Renewable Energy Resources and Electricity Generation

Deployment of reliable, low-carbon, artificial photosynthesis systems is contingent on the availability of renewable energy sources and non-intermittent electricity. Furthermore, achieving protein self-sufficiency from renewable-energy-food-biomass-cultivation systems necessitates an electricity supply that is domestically produced. Iceland is unique in satisfying both these requirements.

**Figure 1:**
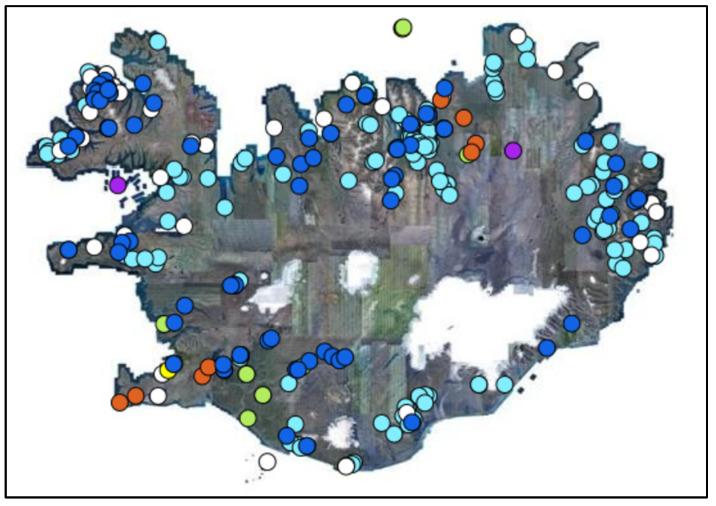
Map of power plants in Iceland. Orange dots represent geothermal power plants, dark blue dots represent hydroelectric power plants, bright green dots represent wind turbine power plants, yellow dots represent solar power plants, purple dots represent petroleum-based power plants, pale blue dots represent home power plants, white dots represent backup power plants. Map data obtained from Government of Iceland [72].

Approximately 85% of the total primary energy supply in Iceland is derived from domestically available and renewable sources. According to government figures, almost 100% of electricity is generated from renewable energy sources, with some 73% derived from hydropower plants and 27% from geothermal power plants, deployed across the island (see Figure 1). On a per capita basis, Iceland is the world’s prime green energy and electricity producer, outputting about 55,000 kWh per person per year. By comparison, in the EU this figure stands at less than 6000 kWh [72]. A historical perspective of how the country achieved its position can be found in previous research [73].

**Figure 2:**
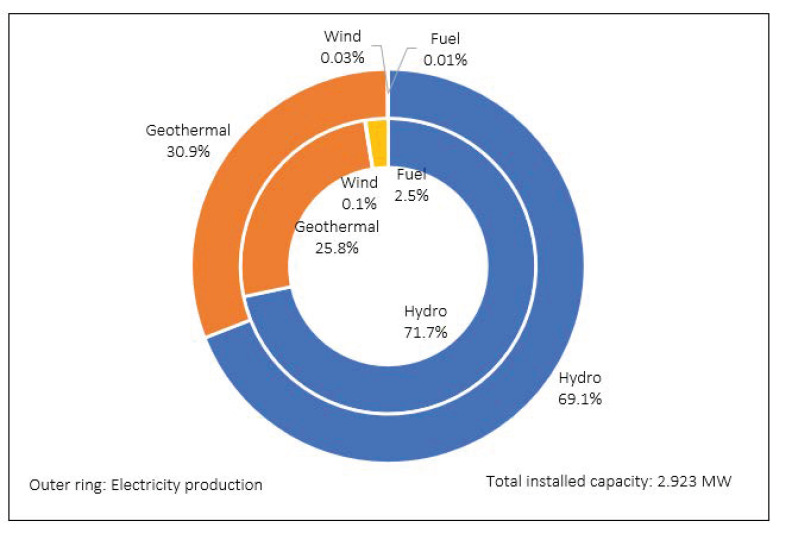
Installed electrical capacity and electricity production in power plants by source in 2019 [74].

According to government statistics on electricity, the total installed capacity stands at 2,923,395 kW, with a total annual production of 19,488,835 MWh. This is mainly produced from hydroelectric (13,461,466 MWh) and geothermal (6,018,138 MWh) sources, together comprising 99.95% of production (see Figure 2).

**Figure 3:**
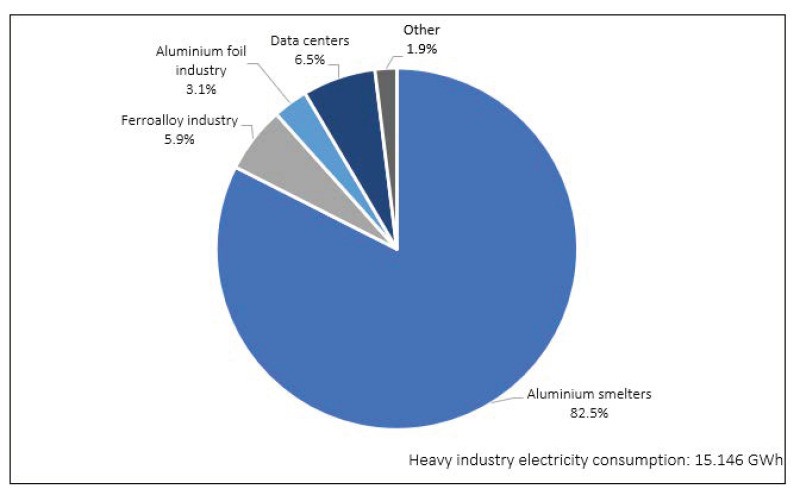
Heavy industry electricity consumption, by category in 2019 [74].

In terms of electricity consumption, conventional users, such as households, make use of 17% (equivalent to 3,375,430 MWh), while heavy industry accounts for 78% (equivalent to 15,146,410 MWh), of total electricity consumption. Within heavy industry, aluminum smelting plants account for 82% of electricity consumed (see Figure 3).

Installed electricity capacity and production in Iceland may be significantly expanded if domestic available renewable resources, currently unexploited, are utilized. This possibility is the focus of Iceland’s Master Plan for Hydro and Geothermal Energy Resources, which identifies geographies of power generation potential. The Plan proposes feasible energy projects for development and compares their economic and environmental impact. In keeping with sustainability standards, the current version of the Master Plan, approved by the government in June 2022, permits the construction of 16 renewable energy power plants, 10 of which are geothermal. These new “permitted” power plants will provide a combined installed capacity of 1299 MW capable of producing 9322 GWh per year. Additionally, another 17 renewable power plants, 13 of which are hydroelectric, may be permitted after future analysis. These new “on hold” power plants would have an aggregate installed capacity in the range of 940 to 967 MW, with production capacity of 7971 to 8066 GWh per year [75].

This study defines scenarios (Section 2.5) for Spirulina production scale-up based on utilization of currently installed and potentially installed, i.e., new permitted and new on hold, power plants. For the latter, the low-end capacity estimate is adopted.

### 2.5. Spirulina Biomass Production Scale-Up Scenarios

Assessing possible production expansion, the protein self-sufficiency model assumes PUs are deployed across Iceland on marginal lands. PUs may be placed in the vicinity of power plants, such as in the case of the Hellisheidi Spirulina cultivation facility, or connected to the national electrical grid. In either option, PUs are powered with renewable energy sources; namely, hydroelectric, geothermal or other, considering Iceland’s green electric generation system.

In terms of cultivating edible Spirulina biomass, the protein self-sufficiency model regards installed electricity capacity as the limiting factor for production scale-up. Other inputs are not deemed restrictive, in view of Iceland’s abundant freshwater resources, relatively low population density of three people per km^2^ and sizable lands of little agricultural value. A stream of CO_2_ for biofixation in PBRs is assumed to be available upon demand, due to technological advancements in carbon capture and storage (known as CCS, or sequestration) [76,77,78,79,80], including exemplar direct air capture (DAC) plants already operational in Iceland [81,82,83]. Residual, underutilized, CO_2_ streams from geothermal power production operations is further available for biofixation [69]. Additionally, fertilizers, namely nitrogen, phosphorus and iron are not considered limiting production factors in this study.

**Figure 4:**
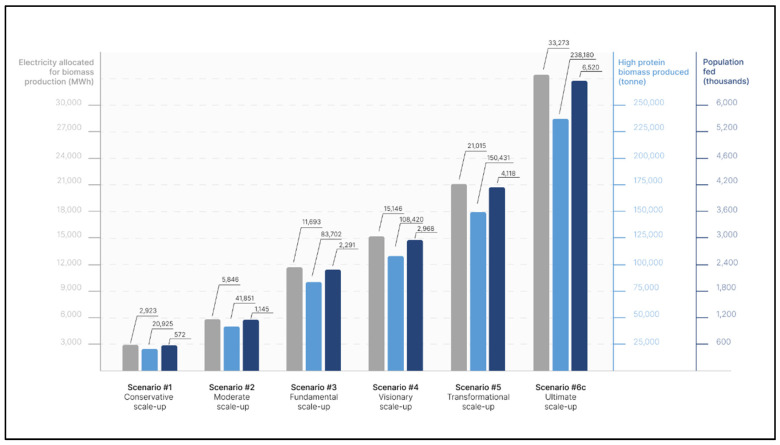
Summary of six high-protein biomass production scenarios as calculated in this study including electricity allocations in MWh, Spirulina biomass production in tonne, and people provided with necessary protein and EAAs in thousands.

Six potential production scale-up scenarios, hypothesizing different electricity allocations, are defined by combining the Spirulina biomass PU definition (Section 2.3) with currently installed and potentially installed electricity generation capacity data (Section 2.4). These scenarios are described below and summarized in Figure 4 and Supplementary Information S1, sheet 1.

#### 2.5.1. Scenario 1: Conservative Scale-Up

The first scenario assumes 15% of current national electricity generation capacity, equivalent to 19% of heavy industry consumption, is allocated to PU operations. This is equivalent to 2,923,325 MWh, which could produce 20,925,736 kg DW Spirulina biomass per year.

#### 2.5.2. Scenario 2: Moderate Scale-Up

In the second scenario, 30% of current national electricity generation capacity (5,846,651 MWh) is allocated for PU operations. This equates to 39% of heavy industry consumption and can support the cultivation of 41,851,471 kg DW Spirulina biomass per year.

#### 2.5.3. Scenario 3: Fundamental Scale-Up

Under a more fundamental scale-up, the third scenario sees 60% of current national electricity generation capacity (11,693,301 MWh), equivalent to 77% of heavy industry electricity consumption, is utilized to produce 83,702,943 kg DW Spirulina biomass per year.

#### 2.5.4. Scenario 4: Visionary Scale-Up

The fourth scenario is visionary in scope and commitment. Here, Iceland allocates 100% of electricity currently consumed by heavy industry, totaling 15,146,411 MWh per year, to produce 108,420,980 kg DW Spirulina biomass per year.

#### 2.5.5. Scenario 5: Transformational Scale-Up

Scenarios 5 and 6 rely on the development of yet to be utilized renewable energy resources as outlined in Iceland’s Master Plan for Hydro and Geothermal Energy Resources.

The fifth scenario assumes that a fundamental 60% of current national electricity generation capacity (as in Scenario 3) plus 100% of new capacity from the 16 renewable-energy power plants permitted for development is exploited for PU operations. This 21,015,301 MWh could yield 150,431,646 kg DW Spirulina biomass per year.

#### 2.5.6. Scenario 6: Ultimate Scale-Up

In the sixth scenario, three options are considered to explore how aspirational Iceland’s Spirulina production scale-up could be in an ultimate utilization of currently installed and potentially installed electricity generation capacity.

In Scenario 6a, a fundamental 60% of current national electricity generation capacity (as in Scenario 3), 100% of new capacity from the 16 renewable energy power plants permitted for development and 100% of new capacity from 17 renewable energy power plants currently on-hold (a combined total of 28,986,301 MWh) are allocated to PU operations. This supports cultivation of 207,489,628 kg DW Spirulina biomass per year.

In Scenario 6b, a transformational reallocation of 100% electricity currently consumed by heavy industry (as in Scenario 4) is combined with 100% capacity of the 33 new renewable energy developments, totaling 32,439,411 MWh to produce 232,207,666 kg DW Spirulina biomass per year.

Finally, in Scenario 6c, Iceland ambitiously maximizes its allocation of current national electricity generation capacity at 82%, in combination with 100% capacity of the 33 new renewable energy power plants currently, to PU operations. This 82% figure assumes that current heavy industry use (78%) and curtailable use (2%) is allocated to Spirulina production, with an additional 2% shaved off conventional use (17%), transmission losses (2%) and distribution losses (1%) through advancements in energy efficiency measures. Of course, the environmental consequences of scenarios 5 and 6a–6c should be carefully evaluated.

### 2.6. Protein Requirements and Demand

Component B of the protein self-sufficiency model integrates various recommended dietary allowance (RDA) values for protein and EAAs, at the individual and population levels, with the nutritional profile of Spirulina cultivated in the Hellisheidi PU, obtained from laboratory analysis. Together, these figures are used to determine the required annual consumption of Spirulina to meet dietary and functional needs.

Laboratory analysis of the Spirulina biomass, conducted by Eurofins Scientific SE, indicates a protein content of 64.95% (0.6495 kg protein per kg DW Spirulina biomass). Table 1 shows the EAAs content of the algae.

Since the Spirulina biomass is cultivated in controlled-environment agriculture (CEA) systems, a consistent nutritional profile can be achieved year-round.

On a mass unit basis (gram of product), Spirulina produced in Hellisheidi PU has higher protein content and higher amounts of EAAs than all ASF, including meat from beef cattle, pigs and poultry, milk and eggs (see Table 2).

When considering the EAAs content on a protein unit basis, conventional meat sources contain similar amounts of EAAs except for lysine and histidine where Spirulina exhibits smaller contents (0.027 kg per kg DW Spirulina biomass and 0.01 kg per DW Spirulina biomass, respectively), and tryptophan where Spirulina shows greater content (0.01 kg per kg DW Spirulina biomass). Milks and eggs have higher amounts of EAAs per gram of protein and are generally considered high-quality protein sources (especially eggs). Nonetheless, Spirulina shows higher amounts of tryptophan compared to eggs and milk.

In terms of satisfactory consumption of proteins to meet all functional needs of adults, 1 g of protein per 1 kg of body weight (BW) is the generally accepted figure [85]. For adult males averaging 70 kg BW and adult females averaging 60 kg BW, this translates to 25.55 and 21.90 kg protein per year, respectively. At a population level, assuming 50:50 women:men ratio, this averages at 23.73 kg protein per person per year.

To ensure sufficient intake of all EAAs prescribed by the FAO, WHO and UNU [86], at a protein content of 64.95%, men would need to consume 39.34 kg DW Spirulina biomass, and women would need to consume 33.72 kg DW Spirulina biomass, per year (see Table 3, and Supplementary Information S1, sheet 2). At a population level, this averages at 36.53 kg DW Spirulina biomass per person per year.

To determine population-wide RDA equivalent demand of protein and EAAs, this study uses United Nations median population estimates and projections for Northern Europe’s countries in 2030 (see Table 4). Sufficient time for production scale-up and construction of PUs and power plants through to 2030 is assumed to satisfy the six scenarios explored in this study.

## 3. Results

### 3.1. Protein Self-Sufficiency Prospects

To assess the feasibility of protein self-sufficiency in Iceland based on industrial production of Spirulina in the novel renewable-energy-food-biomass-cultivation system, as well as to gauge Iceland’s potential role in improving Northern Europe’s protein security, this study integrates model Component A—protein production potential (Section 2.3, Section 2.4 and Section 2.5)—with model Component B—protein consumption requirements (Section 2.6). For each production scale-up scenario, the population whose protein and EAAs requirements can be met is calculated.

#### 3.1.1. Scenario 1: Conservative Scale-Up

In this scenario, an allocation of 2,923,325,268 MWh (15% currently installed capacity) would produce 20,925,736 kg DW Spirulina biomass per year, to meet protein and EAA requirements of 572,867 individuals.

Thus, under this conservative allocation of electricity, Iceland could be protein self-sufficient with a safety margin of close to 50% for its population of 390,338 in 2030. Indeed, Iceland could be protein self-sufficient, with no safety margin, with an allocation of just over 10% currently installed capacity.

One implication of this figure, is that in all following scenarios Iceland is assumed protein self-sufficient as well as a protein net-exporter to Northern Europe’s countries, taking a larger role in regional protein security with each production expansion scenario.

#### 3.1.2. Scenario 2: Moderate Scale-Up

In a moderate allocation of electricity, defined in this study to be 5,846,651 MWh (30% currently installed capacity), some 41,851,471 kg DW Spirulina biomass per year may be manufactured, meeting the protein and EAA requirements of 1,145,734 people.

In this scenario, without developing new energy generation plants, Iceland may feed itself (in terms of protein and EAAs) plus the populations of the British Isles of Jersey, Isle of Man and Guernsey and the Faroe Islands (with respective populations of 119,047, 85,798, 65,141 and 54,583 to give a combined population of 324,569 by 2030), with a remaining excess of biomass for export equating to 430,817 people.

#### 3.1.3. Scenario 3: Fundamental Scale-Up

Assuming a fundamental scale-up, 11,693,301 MWh (60% currently installed capacity) are utilized to produce 83,702,943 kg DW Spirulina biomass per year. This amount should meet the protein and EAA needs of 2,291,467 individuals.

Accordingly, Iceland may be self-sufficient as well as meet the population-wide RDAs of Latvia (population of 1,701,338 by 2030). Alternatively, it may render itself and the entire populations of Estonia (population of 1,289,441 in 2030) plus Jersey, Isle of Man, Guernsey and Faroe Islands protein and EAA secure. This scenario too, does not involve developing new power plants.

#### 3.1.4. Scenario 4: Visionary Scale-Up

In a visionary scenario, 100% of electricity consumed by heavy industry, totaling 15,146,411 MWh per year, is reallocated to PU operation without developing new energy sources. This would support production of 108,420,979 million kg DW Spirulina biomass per year, able to satisfy 2,968,153 people.

This production expansion would see Iceland protein and EAAs self-sufficient with exports adequate to meet the needs of Latvia plus Jersey, Isle of Man, Guernsey and Faroe Islands.

#### 3.1.5. Scenario 5: Transformational Scale-Up

Scenarios 5 and 6 involve the development of new energy resources in addition to utilizing the fundamental (Scenario 3) allocation of 60% currently installed capacity. In the fifth scenario, a total of 21,015,301 MWh is exploited for alternative protein production. This would yield 150,431,647 kg DW Spirulina biomass per year, satisfying 4,118,245 people.

Iceland here is not only self-sufficient but could provide all necessary protein and EAAs for Latvia and Estonia plus Jersey, Isle of Man, Guernsey and Faroe Islands. Alternatively, Iceland could support itself and Lithuania (population 2,558,929 by 2030).

#### 3.1.6. Scenario 6: Ultimate Scale-Up

In the sixth scenario (Scenario 6a), new permitted and on-hold energy generation projects are developed to reach a total of 28,986,301 MWh. This amount would support the manufacture of 207,489,628 kg DW Spirulina biomass per year, meeting the RDAs of 5,680,275 people.

In this significant scale-up, Iceland could satisfy the protein and EAA needs of itself and Ireland (population of 5,266,881 by 2030). Alternatively, Iceland could be self-sufficient as well as support Lithuania and Latvia or Lithuania and Estonia plus Jersey, Isle of Man, Guernsey and Faroe Islands.

Moving from a fundamental (Scenario 3) to transformational (Scenario 4) baseline reallocation of 100% of electricity currently consumed by heavy industry in combination with the new energy generation development would achieve an additional 12% of total energy allocation (Scenario 6b), to reach a total of 32,439,411 MWh. This would provide sufficient electricity to produce 232,207,666 kg DW Spirulina biomass per year, equivalent to protein and EEAs needs of 6,356,960 people.

In this more progressive scale-up, not only will Iceland be self-sufficient, but it could provide for the populations of Finland (population of 5,565,475 in 2030), or Lithuania, Latvia and Estonia, plus Jersey, Isle of Man, Guernsey and Faroe Islands. Otherwise, Iceland could provide sufficient biomass to meet the protein and EEAs requirements of its own population plus Norway (population of 5,748,397 in 2030).

Considering an even more ambitious scale-up (Scenario 6c), requiring 4% efficiency improvements across currently consumed generation, the electricity capacity of the new energy project developments plus 82% of national electricity generation is allocated to PU operation to reach a total of 33,858,510 MWh. This facilitates production of 242,365,854 kg DW Spirulina biomass per year, satisfying 6,635,052 people.

This aspiring scale-up option would see Iceland protein and EAAs self-sufficient with its exports adequate to meet the needs of Denmark (population of 6,104,474 in 2030). Alternatively, Iceland could feed itself as well as Norway plus Jersey, Isle of Man, Guernsey and Faroe Islands.

Figure 4 presents the six scenarios, including electricity allocations, biomass production, and people provided with necessary protein and EAAs.

### 3.2. Global GHG Reduction Potential If Climate Change Mitigation Is Prioritized over Protein Self-Sufficiency

Although not the focus of this feasibility study, it is worth highlighting an ancillary environmental benefit of Spirulina production expansion: the possibility to mitigate global GHG emissions.

Assuming global GHG emissions reduction is prioritized over Northern Europe’s protein self-sufficiency, and instead Spirulina biomass is increasingly and incrementally adopted in Western diets as a beef meat protein alternative (in the form of pills or pressed powder for use as ingredient rather as wholefood), the average consumer may maintain a balanced diet while abating GHG emissions associated with beef cattle agriculture and meat processing.

It has been estimated that the average European consumes nearly 80 kg beef meat per year, while the average North American consumes more than 110 kg beef meat per year [88]. At a GHG intensity of 99.5 kg CO_2_-eq average, personal meat consumption in Europe and North America results in emissions of 7958 and 10,943 kg CO_2_-eq per year, respectively.

Considering overall protein density, an average adult would need to consume 3.17 kg of beef meat (0.205 g protein per g meat) to achieve the same amount of protein available in 1 kg of Spirulina (0.649 g protein per g biomass DW). Therefore, on a protein-per-protein basis, for each kilogram of Spirulina (DW) consumed instead of meat from beef cattle, 315 kg CO_2_-eq could be abated.

To offset the equivalent of 100% of its annual GHG emissions, Iceland would need to produce and consume, as an alternative to beef meat, 9,341,846 kg DW Spirulina biomass. This is less than half the production capacity achievable under the most conservative scale-up (Scenario 1) considered in this study, requiring 1,305,056 MWh, which is equivalent to only 6.7% of currently installed electricity generation capacity [89].

Under the most ambitious Spirulina production scale-up options (Scenario 6a–6c), between 207,489,628 and 238,180,707 kg DW Spirulina biomass per year could be distributed and consumed globally as a beef meat protein alternative. This would translate to a yearly reduction of between 65.4 to 75.1 million metric tonnes CO_2_-eq (MMTCDE).

As a climate change mitigation option, this intervention alone may save 6.4% to 7.3% of Europe’s cross-sector quarterly GHG emissions, currently totaling 1028 MMTCDE (1Q, 2022) [90].

**Figure 5:**
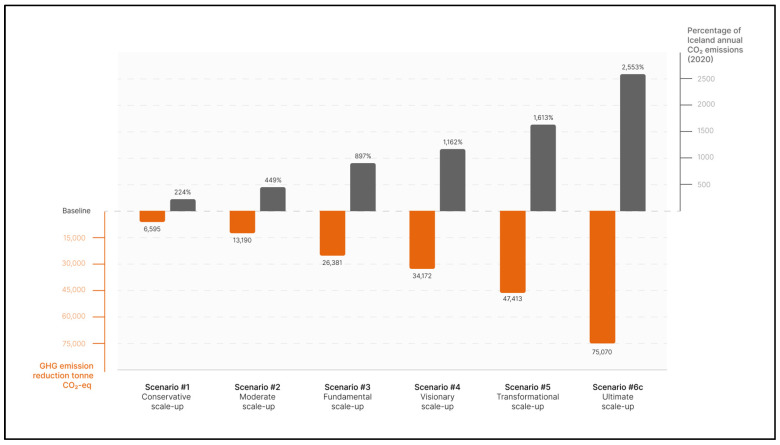
Summary of global GHG emissions reduction potential related to the six Spirulina biomass production scenarios as calculated in this study in CO_2_-eq, including percentage of Iceland’s annual emissions in CO_2_.

Figure 5 presents the six Spirulina production scenarios, global GHG emissions reduction potential for each scenario (CO_2_-eq), and the share of GHG reduced of Iceland’s annual emissions.

## 4. Discussion

### 4.1. Reflection on Key Findings

The results of this feasibility study highlight the promising and tangible potential of large-scale Spirulina production using a novel renewable-energy-food-biomass-cultivation system. With only a conservative allocation of current electricity generation, Iceland could be self-sufficient when it comes to protein and EEAs, using Spirulina as an alternative protein source that is nutritious, sustainable and risk resilient. Additionally, with large-scale production scale-up, utilizing both current and planned energy assets, Iceland could similarly help reduce its Northern European neighbors’ dependency on high-protein food crop and traditional ASF imports, addressing a hot topic on the policy agenda.

In the process, there is an opportunity for Iceland revolutionize its industry. By reallocating electricity currently consumed by heavy industry, it could transition to a position as a major and sustainable alternative protein exporter.

Beyond the focus of this study on its high-density protein content (~65%) and complete EEAs profile, Spirulina offers further nutritional advantages. The algae also contain the other macro-nutrients of carbohydrate (24%) and fats (8%) for an energy content of 1213 kJ per 100 g. It is also rich in essential micro-nutrients, including B vitamins, such as Thiamine and Riboflavin, and dietary minerals, such as iron (Fe) and manganese (Mn) and magnesium (Mg). Moreover, in addition to the associated GHG abatement potential that the net-negative emissions production, the cultivation of Spirulina engenders other sustainability benefits. On a functional unit (1 kg) basis, the production of Spirulina, especially in the stackable, multicompartment PUs modelled in this study, results in less than 1% of the land and water footprint of meat from beef cattle and other ASF [43].

Furthermore, the production of Spirulina in the configuration proposed in this study, provides the opportunity to achieve risk resilient diets. Specifically, the PBR-based production system modeled provides a closed and controlled cultivation environment, which reduces exposure to biotic and abiotic risk factors that otherwise jeopardize traditional plant-source food (PSF) and ASF agriculture. The modular design that can be adopted with such PUs also enables flexible and agile response to unexpected disruptions as well as efficiency in planned maintenance and adjustment of production to oscillating demand [32].

Finally, as opposed to previous, assumption-based modelling studies, for both open- and closed- aquaculture systems [91,92,93], this feasibility assessment draws on data retrieved from an actual, large-scale, and fully operational PU, currently manufacturing protein-dense Spirulina biomass in Iceland. Accordingly, this analysis comparatively suffers little uncertainty. Nonetheless, there are limitations to the interpretation of this study, discussed below.

### 4.2. Reservations

First, it is important to note that the results presented here indicate the absolute number of people whose protein requirements could theoretically be met by the production of Spirulina; the results do not indicate actual uptake of Spirulina by a given population, as this would introduce speculative consideration of uncertain dietary and pricing preferences into an otherwise robust technical calculation.

Protein self-sufficiency and environmental benefits may only be realized if consumers choose to adopt Spirulina into their diets, including daily meals. While this matter still requires further research, which is outside the scope of this investigation, existing research into food neophobia and consumer acceptance of Spirulina as a protein alternative indicated that health-conscious consumers, including vegetarians, foodies and sporting individuals are willing to integrate the algae into their menus [94,95,96].

Similarly, consumers must be able to afford Spirulina as a staple source of protein for these benefits to be realized. Research into Spirulina market pricing remains underdeveloped, however, spot pricing in the UK at Sainsbury’s supermarkets for Spirulina is approximately £74 per kg of protein (£5.00 for a 100 g packet of Spirulina at 67 g protein per 100 g) while lean beef mince is approximately £20 per kg of protein (£3.20 for a 500 g packet of 5% fat beef mince at 31.3 g protein per 100 g). While Spirulina is currently less affordable than beef, it may be speculated that Spirulina would converge to a similar price point of conventional protein sources once it similarly becomes produced at mass-scale.

Second, an economic analysis of capital and operating costs as well as product pricing specific to the Hellisheidi PU is outside the scope of this study, and there is currently a lack research on the financial feasibility of Spirulina as a major source of protein more broadly. As such, it is important to bear in mind the finance necessary to ramp-up Spirulina production, including that required for construction of PU infrastructure as well as investments necessary for training a new labor force and educating the public about consumption benefits. The availability, or lack thereof, of both financial and human capital may hinder the realization of this protein self-sufficiency model.

However, funding constraints may be addressed in one of two strategies into the future. First, Iceland could design alternative protein supply contracts to lock in exportation quantities, in a similar way to which aluminium trade is handled. Second, the GHG abatement potential of Spirulina production opens an additional revenue stream through the sale of carbon credits. With a global average price of $2.49 per tonne CO_2_-eq on the voluntary market in 2022, Iceland could issue between $16.4 million (Scenario 1) to $187 million (Scenario 6c) of carbon credits per year.

Furthermore, beyond government and other domestic funding, which may prove substantial given the industry transition up for grabs, the advantages and disadvantages of private equity and venture capital, particularly given the rapid rise of sustainable finance and venture buzz in the alternative protein and climate technology space, to bolster the large-scale production of Spirulina should be considered.

Thirdly, the Spirulina production scale-up necessary for Iceland to meet its own, as well as Northern Europe’s, protein and EEAs requirements is dependent on the allocation of adequate electricity. In reality, this allocation may not be feasible due to competing demands, including existing residential, commercial and industrial uses as well as demand from other forms of traditional and non-traditional agriculture. For instance, while the 100% reallocation of electricity used by heavy industry to Spirulina production has promise, the economic transition outlined above (Section 4.1) would surely be met with opposition by the aluminium sector. Similarly, the 100% allocation of all new in Iceland’s Master Plan would be an ambitious undertaking, likely with political complexity.

## 5. Conclusions

In response to growing concerns about global food security, specifically reliance on imports for conventional protein source feed in Europe, and growing interest in alternative protein sources as a potential solution, this study explored six scenarios of high-protein, high-EAA Spirulina production based on scale-up of a currently operating cultivation unit, powered by renewable energy, in Iceland, with the aim of contributing to the growing literature on protein self-sufficiency in Europe.

Firstly, this investigation sought to assess the feasibility of protein self-sufficiency in Iceland. It found that with approximately 10% of currently installed electricity generation dedicated to Spirulina production, Iceland could be protein and EEAs self-sufficient. Secondly, this feasibility assessment explored Iceland’s role in supporting Northern European countries to achieve protein self-sufficiency through further scale-up of its Spirulina production capacity and export of the edible biomass. In the most ambitious scenario, prioritizing Spirulina as primary industry and dedicating all future energy developments to biomass cultivation, Iceland could be self-sufficient plus provide exports meeting the protein and EEAs needs of over 6 million Northern Europeans.

Furthermore, the novel renewable-energy-food-biomass-cultivation system modeled in this study is net-negative in terms of GHG emissions. As a result, the production of Spirulina powered by 10% of currently installed electricity generation would abate 6.5 million tonnes CO_2_-eq, while the most ambitious scenario would reduce some 75 million tonnes CO_2_-eq.

However, the scenarios considered here assume idealized production conditions, and prospects for transitioning food systems should consider financial and non-financial issues that may pose barriers to realizing the opportunity highlighted by the protein self-sufficiency model in this study. Specifically, to improve our understanding of the potential of Spirulina as an alternative protein source, future research could consider underexplored aspects of consumer dietary preferences, consumer affordability, capital and operating expenses and financing opportunities. Additionally, future research could perform similar feasibility studies for other alternative protein sources, such as microbial cultures, in isolation or combination to further contribute to our understanding of their role in supporting protein self-sufficiency.

## Figures and Tables

**Table 1:** Protein and EAAs content of Spirulina biomass DW produced in Hellisheidi PU.

Content	Value	Unit
Protein	0.6495	kg protein per kg DW Spirulina biomass
Threonine	0.0307	kg EEA per kg DW Spirulina biomass
Valine	0.0335	kg EEA per kg DW Spirulina biomass
Isoleucine	0.0351	kg EEA per kg DW Spirulina biomass
Leucine	0.0538	kg EEA per kg DW Spirulina biomass
Phenylalanine	0.0293	kg EEA per kg DW Spirulina biomass
Lysine	0.0279	kg EEA per kg DW Spirulina biomass
Histidine	0.0100	kg EEA per kg DW Spirulina biomass
Methionine	0.0141	kg EEA per kg DW Spirulina biomass
Tryptophan	0.0097	kg EEA per kg DW Spirulina biomass

**Table 2:** Protein content of Spirulina and selected animal source foods (ASF). Values for ASF in this table are obtained from National Food Institute, Technical University of Denmark [84].

Food Source	Protein Content	Unit
Spirulina (Hellisheidi PU)	0.649	g protein per g product
Meat, beef	0.205	g protein per g product
Meat, lamb	0.196	g protein per g product
Meat, pig	0.212	g protein per g product
Meat, poultry	0.215	g protein per g product
Eggs	0.123	g protein per g product
Milk	0.034	g protein per g product

**Table 3:** Required intake of Spirulina biomass to meet recommended dietary allowance of protein and EAA, men and women.

**Men (Avg. BW 70 kg)**	**Amount**	**Unit**
Spirulina to meet protein requirement	39.33	kg DW Spirulina biomass per year
Spirulina to meet threonine requirement	12.46	kg DW Spirulina biomass per year
Spirulina to meet valine requirement	19.80	kg DW Spirulina biomass per year
Spirulina to meet isoleucine requirement	14.55	kg DW Spirulina biomass per year
Spirulina to meet leucine requirement	18.50	kg DW Spirulina biomass per year
Spirulina to meet phenylalanine requirement	21.76	kg DW Spirulina biomass per year
Spirulina to meet lysine requirement	27.47	kg DW Spirulina biomass per year
Spirulina to meet histidine requirement	25.32	kg DW Spirulina biomass per year
Spirulina to meet methionine requirement	18.12	kg DW Spirulina biomass per year
Spirulina to meet tryptophan requirement	10.53	kg DW Spirulina biomass per year
**Women (avg. BW 60 kg)**	**Amount**	**Unit**
Spirulina to meet protein requirement	33.71	kg DW Spirulina biomass per year
Spirulina to meet threonine requirement	10.68	kg DW Spirulina biomass per year
Spirulina to meet valine requirement	16.97	kg DW Spirulina biomass per year
Spirulina to meet isoleucine requirement	12.47	kg DW Spirulina biomass per year
Spirulina to meet leucine requirement	15.86	kg DW Spirulina biomass per year
Spirulina to meet phenylalanine requirement	18.65	kg DW Spirulina biomass per year
Spirulina to meet lysine requirement	23.54	kg DW Spirulina biomass per year
Spirulina to meet histidine requirement	21.70	kg DW Spirulina biomass per year
Spirulina to meet methionine requirement	15.53	kg DW Spirulina biomass per year
Spirulina to meet tryptophan requirement	9.03	kg DW Spirulina biomass per year

**Table 4:** Population estimates and projections (2030) in Northern Europe [87].

Country	Population Projections (2030)
United Kingdom	69,175,770
Sweden	11,007,228
Denmark	6,104,474
Finland	5,565,475
Norway	5,748,397
Ireland	5,266,881
Lithuania	2,558,929
Latvia	1,701,338
Estonia	1,289,441
Iceland	390,338
Jersey	119,047
Isle of Man	85,798
Guernsey	65,141
Faroe Islands	54,583

## Data Availability

Data and materials used in this study are fully delineated in the text, Supplementary Information (SI) and reference list.

## Supplementary Materials

The following supplementary information can be downloaded at: https://www.mdpi.com/article/10.3390/foods12010038/s1, Supplementary Information (SI S1) consists of the “protein self-sufficiency model” available as an Excel spreadsheet.
